# Comparative Study on the Wear Resistance of C&B-Type Polymer Materials for Temporary Crowns Manufactured Using 3D DLP Printing Technology

**DOI:** 10.3390/ma18245478

**Published:** 2025-12-05

**Authors:** Marcel Firlej, Daniel Pieniak, Andrzej Snarski-Adamski, Barbara Biedziak, Agata Niewczas, Jana Petru, Jonas Matijošius, Zbigniew Krzysiak, Katarzyna Zaborowicz

**Affiliations:** 1 Department of Craniofacial Anomalies, Poznan University of Medical Sciences, Bukowska 70, 60-812 Poznan, Poland; marcel-firlej@wp.pl (M.F.); biedziak@ump.edu.pl (B.B.); kzaborowicz@ump.edu.pl (K.Z.); 2 Tribology Center, Łukasiewicz Research Network-Institute for Sustainable Technologies (L-ITEE), Pułaskiego 6/10, 26-600 Radom, Poland; andrzej.snarski@itee.lukasiewicz.gov.pl; 3 Department of Conservative Dentistry with Endodontics, Medical University of Lublin, W. Chodźki 6, 20-093 Lublin, Poland; agata.niewczas@umlub.pl; 4 Faculty of Mechanical Engineering, VSB—Technical University of Ostrava, Poruba, 70800 Ostrava, Czech Republic; jana.petru@vsb.cz; 5 Mechanical Science Institute, Vilnius Gediminas Technical University, Plytinės Str. 25, 10105 Vilnius, Lithuania; 6 Department of Mechanical Engineering and Automation, Faculty of Production Engineering, University of Life Sciences in Lublin, Głęboka 28, 20-612 Lublin, Poland; zbigniew.krzysiak@up.lublin.pl

**Keywords:** DLP 3D printing, temporary crowns, dental polymers, wear resistance, scratch resistance, filler content

## Abstract

**Highlights:**

**What are the main findings?**

- VarseoSmile Temp had the lowest wear and highest scratch resistance.
- Wear resistance depends on the amount of filler and microstructure.
- No direct relationship was found between the coefficient of friction and wear.
- Higher inorganic filler content improved surface elasticity.

**What is the implication of the main findings?**

- VarseoSmile Temp is best suited for temporary crowns with long-term stability.
- Tribological testing allows for the prediction of clinical behavior of 3D printed materials.
- Optimization of the filler improves functional and durability indicators.
- Test results in a humid, 37 °C environment reliably reflect oral conditions.

**Abstract:**

DLP (Digital Light Processing) 3D printing enables precise fabrication of temporary crowns. Tribological properties of these materials affect clinical durability, wear resistance, and masticatory function. This study compared three C&B-type photopolymers for DLP-printed temporary crowns: Gr-17.1 temporary It, Gr-17 temporary (Pro3dure), and VarseoSmile Temp (BEGO). Samples were printed, post-processed, and polished. Surface topography (Sa, Sz) was measured via white light interferometry, and scratch resistance was evaluated with a Rockwell indenter. Sliding wear tests under wet conditions (37 °C, 90% RH) were conducted using an SRV 4 tester at 25 N for 20,000 cycles. VarseoSmile Temp showed the highest scratch and sliding wear resistance, with the lowest mean volumetric wear (0.025 mm^3^) and residual scratch depth, reflecting its higher inorganic filler content (30–50 wt%). Gr-17.1 had the most stable coefficient of friction (~0.3), while Gr-17 experienced the greatest wear (0.235 mm^3^). No direct correlation between friction and wear was observed. These findings indicate that wear resistance depends on microstructure and filler content, supporting tribological testing as a tool to evaluate the durability of 3D-printed temporary crowns.

## 1. Introduction

Provisional restorations provide a crucial protective and diagnostic role in dental therapy, ensuring teeth alignment, safeguarding periodontal tissues, facilitating masticatory function, and enhancing smile aesthetics [1,2]. Temporary crowns are needed for extended prosthetic rehabilitations, rapid implant loading, and gingivectomy surgeries, with functional periods ranging from several months to even longer [3,4,5].

The wear of dental materials is a natural process resulting from contact and friction between opposing surfaces in the oral cavity. This process includes abrasive, adhesive, and fatigue phenomena, which may occur individually or in combination, along with synergistic effects such as tribochemical corrosion [6,7,8,9]. In dentistry, tribological wear—also known as attrition—is the most common cause of degradation of both dental tissues and prosthetic restorations [10,11]. The intensity of this wear is determined by occlusal pressures, surface geometry, and the presence of wear debris [12,13]. Abrasive wear occurs when the contacting surfaces are rough or when abrasive particles are present in the contact zone [14,15]. For hard and brittle materials, surface asperities may deform or fracture, whereas in the case of significant hardness differences between antagonistic materials, microscratching and accelerated material loss can occur [8].

Tribological research has demonstrated that the wear process of crown materials occurs in specific stages. During the first phase, characterized by low frictional forces in relation to the normal load, Hertzian contact mechanics prevail, leading to subsurface stress concentration at the Hertz/Bielajew point [16]. When the friction coefficient goes up, the largest stresses move toward the surface, and critical stress concentrations arise outside the contact zone, which causes the surface layer to flex plastically [17]. Subsequent friction cycles facilitate the coalescence of microcracks into larger fissures, which, under higher friction conditions, may lead to material fracture. Clinical investigations indicate that the loss of occlusal height in molar crowns was 0.47 ± 0.70 mm after 12 months and 0.76 ± 1.06 mm after 24 months [18,19,20].

The wide range of these data highlights the significant impact of unique patient features, such as cyclic occlusal loads, necessitating tribological testing approaches that incorporate varied load levels and scenarios [21].

Digital dentistry and 3D printing technologies, particularly Digital Light Processing (DLP), are advancing rapidly. This allows you to create temporary crowns and bridges with complex shapes and the greatest material combinations [22,23,24]. The clinical efficacy of prosthetic restorations is contingent upon the material characteristics, the extent of monomer-to-polymer conversion, and the caliber of surface finishing [25,26]. Post-processing techniques, including as washing and further UV curing, enhance polymerization and surface integrity in printed crowns [27]. Surface resistance to wear and scratching is a key factor determining the durability of dental materials; its degradation may result from microscratching, surface creep, or tribofilm formation and transformation [28,29,30].

Polymeric crown wear can be either physiological or pathological in nature. Under bruxism conditions, occlusal forces may reach up to 650 N, leading to localized deformation of crown and bridge materials [31,32,33,34,35]. In three-body wear scenarios—such as the presence of a food–saliva suspension—microscratches and exposure of filler particles within the polymer matrix are commonly observed [12,36]. This process happens slowly over time. At first, the load is distributed over larger bits of food, and later, small particles of suspension penetrate the surface asperities, making them rougher and less scratch-resistant [29,30].

According to the current state of knowledge, tribological evaluation of polymer-based dental materials is essential for assessing their service durability, predicting the wear behavior of temporary crowns, and designing materials with optimized surface resistance, ensuring both functional performance and patient safety [17].

The aim of this study was to perform a comprehensive comparative evaluation of three photopolymer-based C&B materials intended for the fabrication of temporary crowns using DLP technology, with particular emphasis on the material characteristics that determine their clinical durability. The investigation focused on microstructural features and surface topography after finishing procedures; microhardness; scratch resistance under single-step and cyclic loading, and tribological behavior under simulated intraoral conditions (37 °C, high humidity), including friction coefficient stability and volumetric wear. By analyzing these parameters in parallel, the study sought to determine the influence of filler content and microstructural composition on the mechanical and tribological performance of the tested materials, and to provide a basis for predicting their behavior under masticatory loading during clinical use.

## 2. Materials and Methods

Three commercially available C&B (crown and bridge) materials based on polymer resins and designed for 3D printing using the Digital Light Processing (DLP) technology were used in this study: Gr-17.1 temporary It and Gr-17 temporary (Pro3dure, Iserlohn, Germany), as well as VarseoSmile Temp (Bego, Bremen, Germany). The main characteristics of the tested materials are summarized in Table 1.

Disc-shaped specimens (30 mm in diameter and 8 mm in height) were printed according to the manufacturers’ recommendations. The 3D printing of the materials was carried out using an ASIGA MAX UV DLP 3D printer (Asiga, Sydney, Australia) with a light source wavelength of 385 nm. The printing AI factory software parameters included a heater temperature of 40 °C, light intensity of 6, 1 burn-in layer, and a burn-in exposure time of 0.5 s. The samples were rinsed in 99.9% isopropyl alcohol for 6 min, then dried and UV-cured for 4 min with an Anycubic All-in-One Wash & Cure equipment. Surface finishing included sequential grinding with SiC abrasive papers of P600, P1200, and P2400 grit sizes, followed by polishing on an automatic grinding and polishing machine Saphir 550 (ATM GmbH, Bielefeld, Germany) using a textile disc and a 1 µm diamond suspension.

Surface roughness after polishing was evaluated using a Taylor Hobson Talysurf CCI optical profilometer based on the principle of white light interferometry (WLI). The device allows for three-dimensional surface mapping. In accordance with ISO 25178 [42], the surface topography parameters were determined: Sa—arithmetic mean height, and Sz—maximum height of the 3D surface profile.

Microhardness test was performed using the Vickers hardness test method. In this method, a square-based pyramid diamond indenter with face angles of 136° degrees is impressed into the surface of the test specimen. The tests were conducted using a Futertech FM 800 (Future-Tech Corp., Kawasaki-City, Japan) apparatus. The load used was 300 g and the penetration time was 20 s. Measuring coordinates were set to cover the entire surface area of a specimen and were the same for all specimens. The analysis area was 6 mm × 6 mm.

Scratch resistance tests were performed using a Micro Scratch Tester (MST, Anton Paar GmbH, Ostfildern-Scharnhausen, Germany). Scratches were made with a Rockwell-type indenter (diamond cone with a tip radius of 100 µm) (Buehler, Echterdingen, Germany). The applied normal load increased stepwise from 1 N to 5 N, with a sliding speed of 1 mm/min and a total scratch length of 1 mm. Additionally, cyclic scratch resistance tests were carried out by performing ten linear scratches under a constant normal load of 0.8 N, a sliding speed of 1.6 mm/min, and a total length of 0.8 mm. Scratch morphology, residual depth (Rd), penetration depth (Pd) and evidence of plastic deformation or filler pull-out were analyzed. The procedure for determining the Pd and Rd parameters was as follows: first stage: determination of surface profile—the depth recorded during the Prescan phase, representing the initial topography of the specimen surface prior to loading; second stage: determination of Pd (penetration depth)—the depth measured during the scratch phase, calculated as the instantaneous difference between the scratch trajectory and the Prescan profile. This value reflects the total deformation of the material under the applied load (elastic + plastic components); third stage: determination of Rd (residual depth)—the difference between the Postscan and Prescan profiles, representing the permanent deformation remaining after unloading. This parameter quantifies the plastic component of the scratch response.

**Figure 1:**
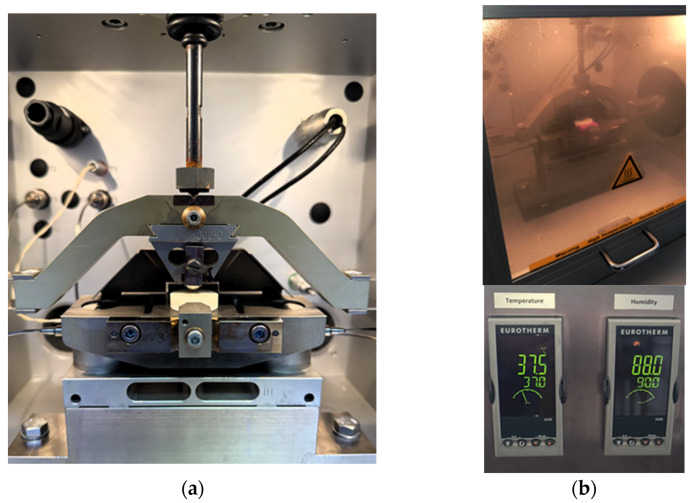
Tribological testing setup: (**a**) test configuration—ball (Ø10 mm, bearing steel 100Cr6) against disc (specimen made of tested material, Ø30 mm); (**b**) test performed under simulated physiological conditions (relative humidity 90%, temperature 37 °C).

Tribological tests were conducted under oscillating motion using an SRV 4 tribometer (Optimol Instruments Prüftechnik GmbH, Munich, Germany) equipped with a climatic chamber maintaining a temperature of approximately 37 °C and relative humidity up to 90% (Figure 1), to replicate physiological intraoral conditions. The specimens were mounted in a fixed holder and subjected to a normal load of 25 N for 20,000 cycles, following the procedure described by [43]. A 10 mm diameter bearing steel ball oscillated against the specimen surface at a frequency of 2 Hz [44].

## 3. Results

**Figure 2:**
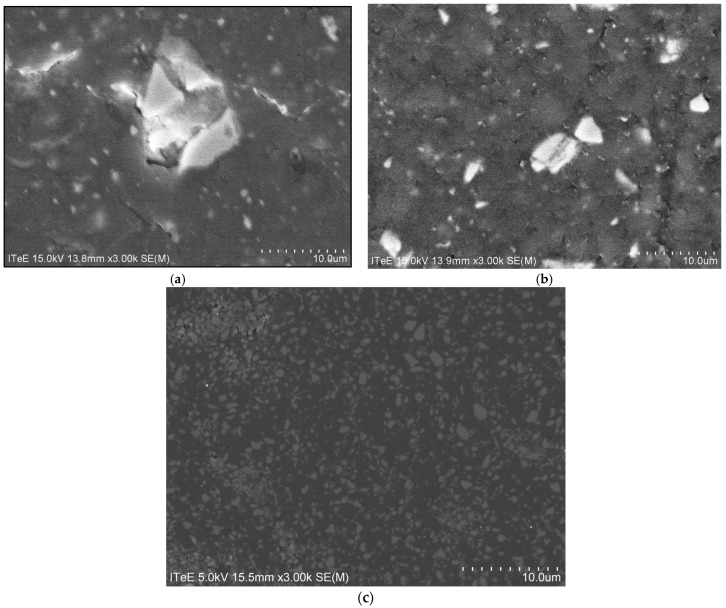
SEM images of tested materials structures: (**a**) Gr-17.1, (**b**) Gr-17, (**c**) VST.

The structure of the samples is presented in Figure 2 in the form of SEM images taken for the 30 µm × 40 µm area.

**Figure 3:**
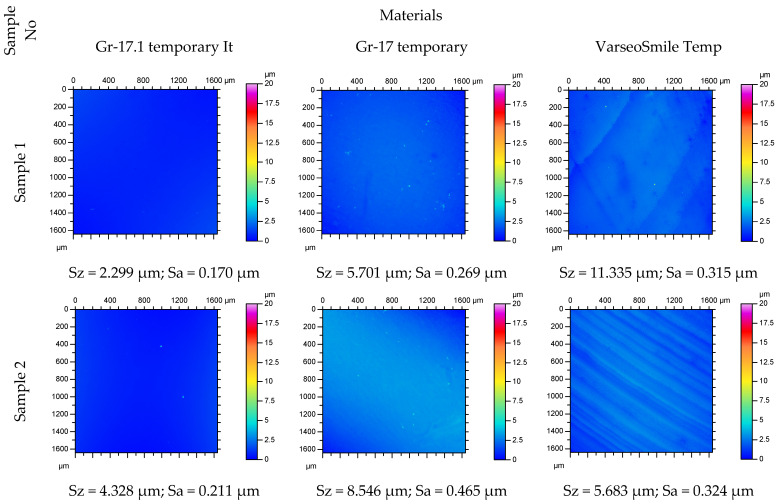

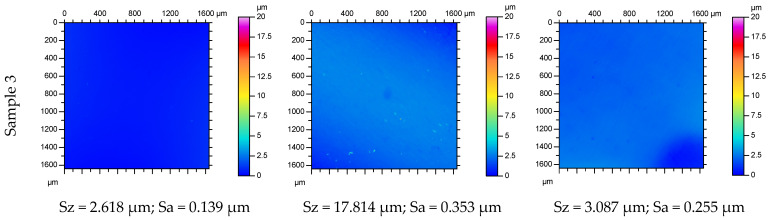
Surface topography of the tested material samples.

Surface topography after polishing are presented in Figure 3. The profilograms show the surface condition of selected samples on a 1.6 mm × 1.6 mm surface after printing and after grinding and polishing. Clear differences between the materials are visible.

**Figure 4:**
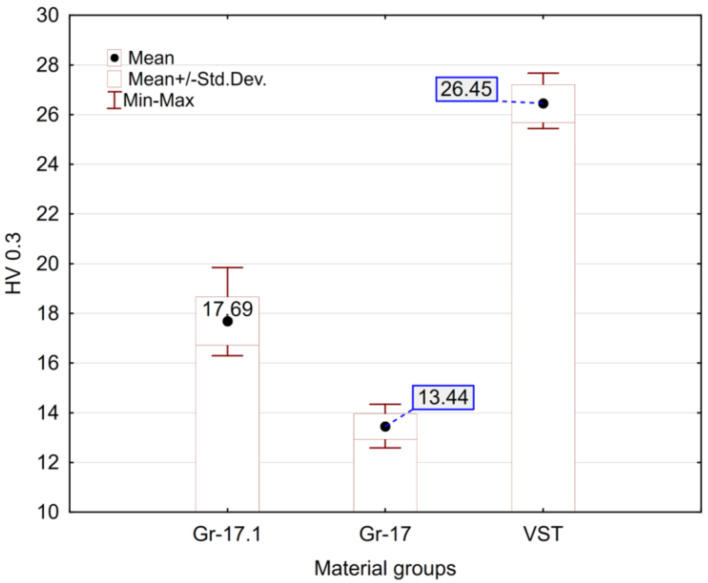
Microhardness of the tested material samples.

The microhardness test results are presented in Figure 4. The hardness values of the tested materials differ substantially. The hardest material was VST, while the lowest hardness was recorded for Gr-17, whose average value was approximately half that of VST.

**Figure 5:**
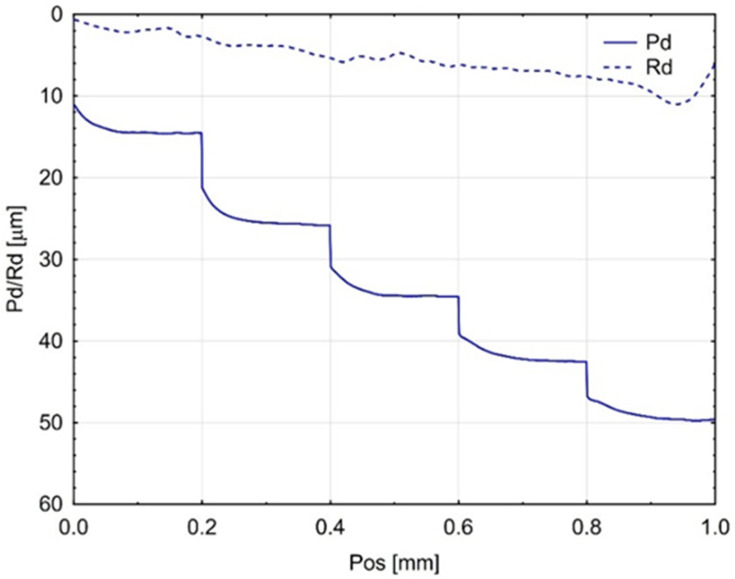
Mean curves (from three Gr-17.1 samples) of indenter penetration depth under test load (Pd) and residual scratch depth (Rd).

**Figure 6:**
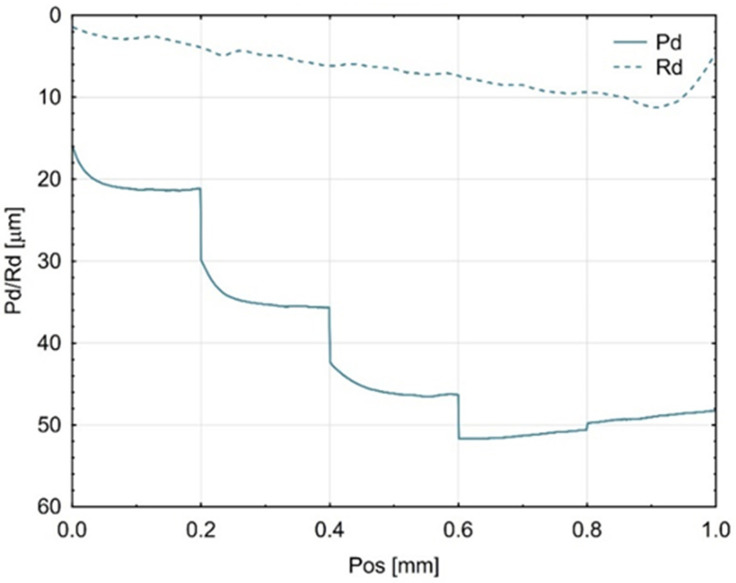
Average curves (from three Gr-17 material samples) of indenter penetration depth under test load (Pd) and permanent groove depth (Rd).

**Figure 7:**
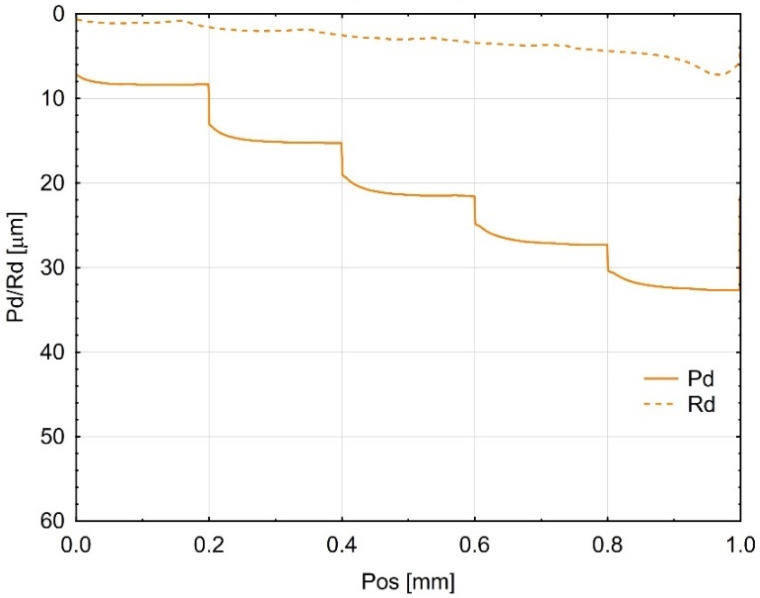
Average curves (from three VST material samples) of indenter penetration depth under test load (Pd) and permanent scratch depth (Rd).

The incremental scratch test (step-loading method) provided values for the residual scratch depth (Rd) and the penetration depth (Pd), as shown in Figure 5, Figure 6 and Figure 7. The penetration depth (Pd) differed significantly from the residual scratch depth (Rd) for all tested materials. The Pd value increased proportionally with the incremental test load; however, for the Gr-17 material, Pd did not increase at the last two load levels. This behaviour under the applied indenter load did not correspond to any notable change in the shape of the Rd curve.

The greatest residual scratch depth under the maximum load (5 N) was observed for the Gr-17 material, with a comparable depth recorded for Gr-17.1.

This indicates that the Gr-17 resin exhibits lower elastic recovery and higher plastic deformation under localized stress, which may be attributed to its lower filler content and higher polymer matrix flexibility. The VST material exhibited a reduced difference between Pd and Rd, indicating an enhanced capacity for elastic recovery and superior resistance to permanent deformation. The results indicate that the mechanical response observed during indentation is significantly affected by the microstructure and filler composition of the resins tested, which in turn influences their scratch resistance and overall surface durability.

**Figure 8:**
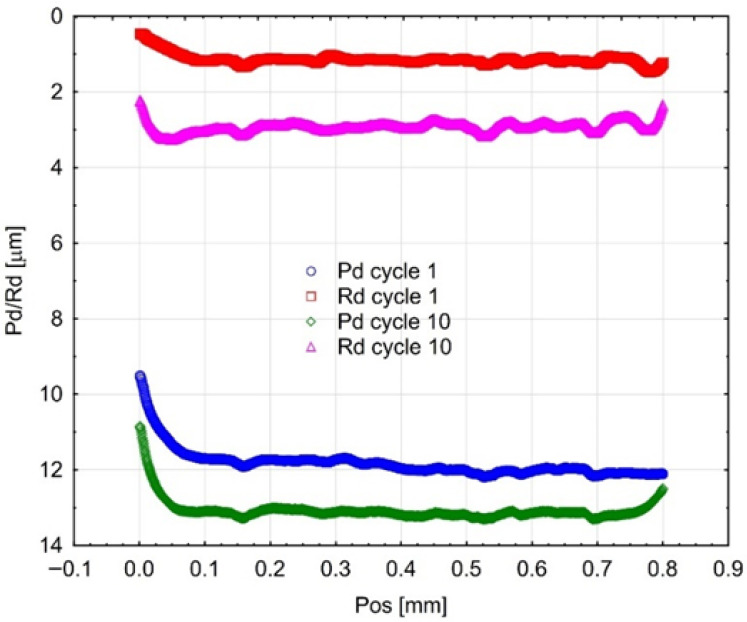
Mean curves (3 samples of each material) describing the course of cyclic scratching on the surface of the material sample Gr-17.1.

**Figure 9:**
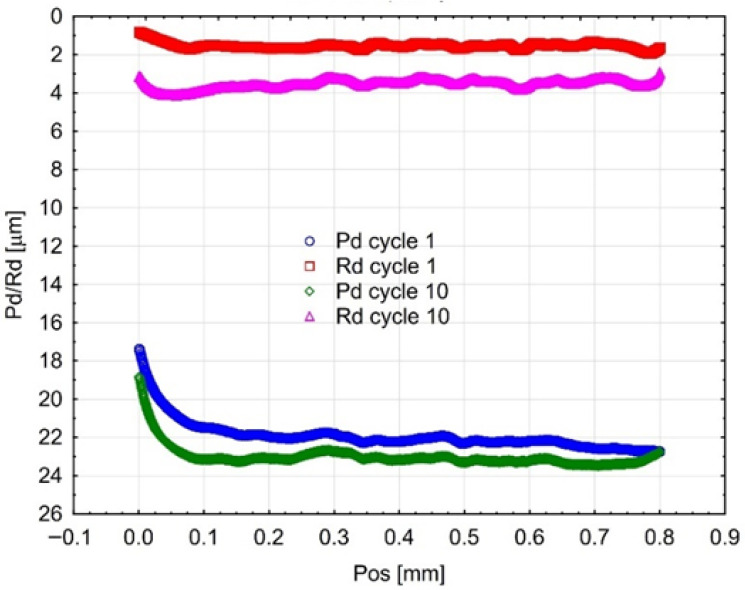
Mean curves describing the course of cyclic scratching on the surface of the material sample Gr-17.

**Figure 10:**
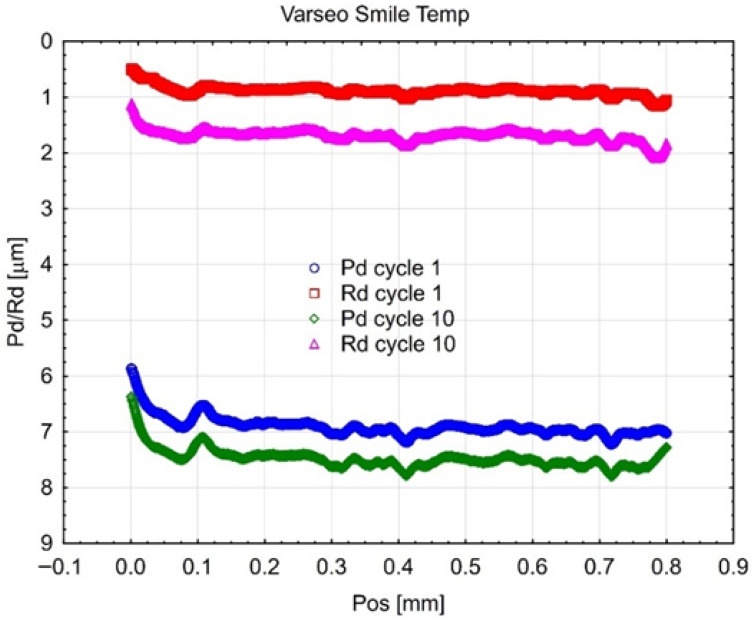
Mean curves describing the course of cyclic scratching on the surface of the material sample VST.

Figure 8, Figure 9 and Figure 10 present the results of cyclic scratch resistance tests. Curves for both parameters are shown for the first and the tenth (final) cycle of the test.

The Gr-17 material exhibited the maximum residual scratch depth (Rd) and penetration depth (Pd) under the applied load. The Rd values for Gr-17.1 and Gr-17 exhibited less variability compared to the Pd values. The variation in response of the two materials to cyclic loading indicates distinct elastic recovery properties.

The VST material demonstrates outstanding durability against repeated abrasion. The Rd values measured after the initial and tenth cycles were the lowest among all tested materials, and the Pd values exhibited a similar reduction.

The results show that the VST resin has better surface resilience and better resistance to cumulative plastic deformation after being loaded multiple times. This behavior can be ascribed to a more homogeneous distribution of fillers and an increased crosslinking density within the polymer matrix. It is crucial for the long-term efficacy of temporary crowns that are subjected to repeated occlusal loading that VST maintains mechanical integrity under cyclic stress, as evidenced by the reduced increase in Rd and Pd across consecutive cycles.

**Figure 11:**
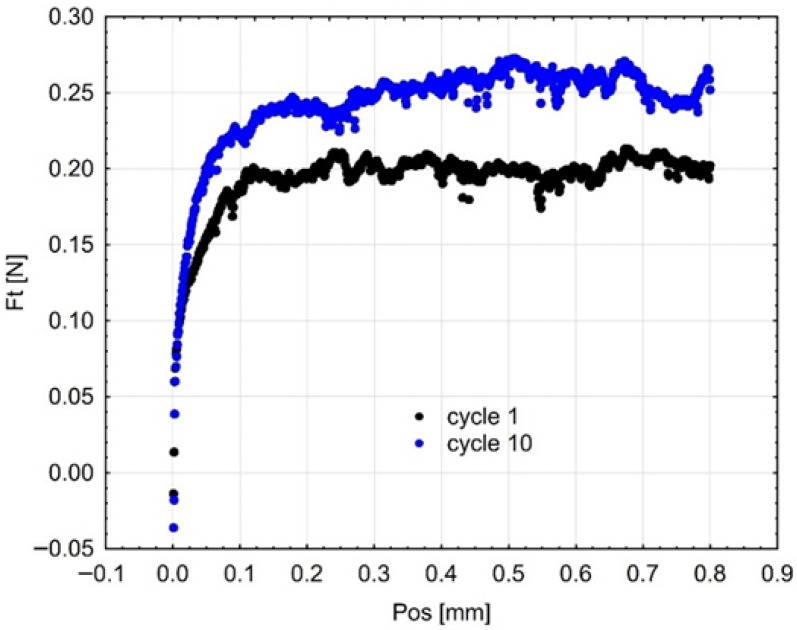
Mean curves (3 samples of each material) describing the course of cyclic scratching on the surface of the material sample Gr-17.1.

**Figure 12:**
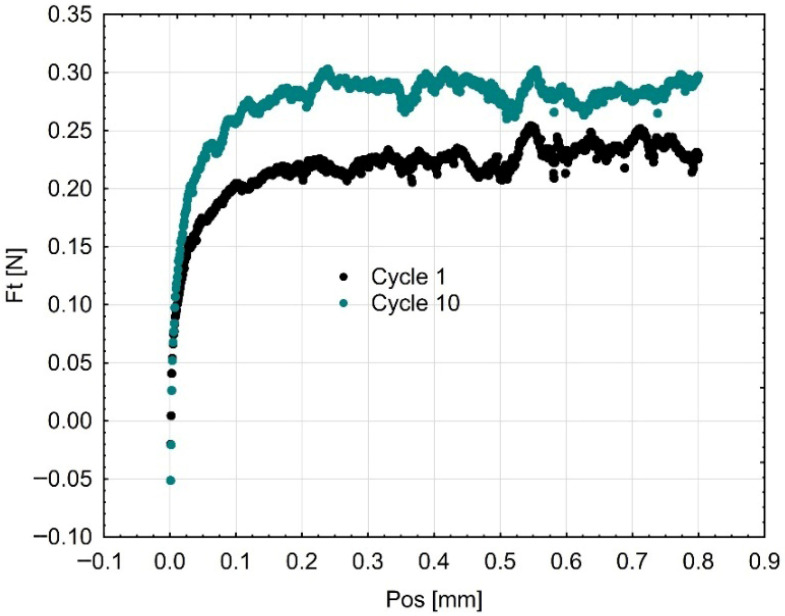
Resistance to scratching (friction force) in the first and tenth cycles on the surface of the Gr-17 temporary material sample.

**Figure 13:**
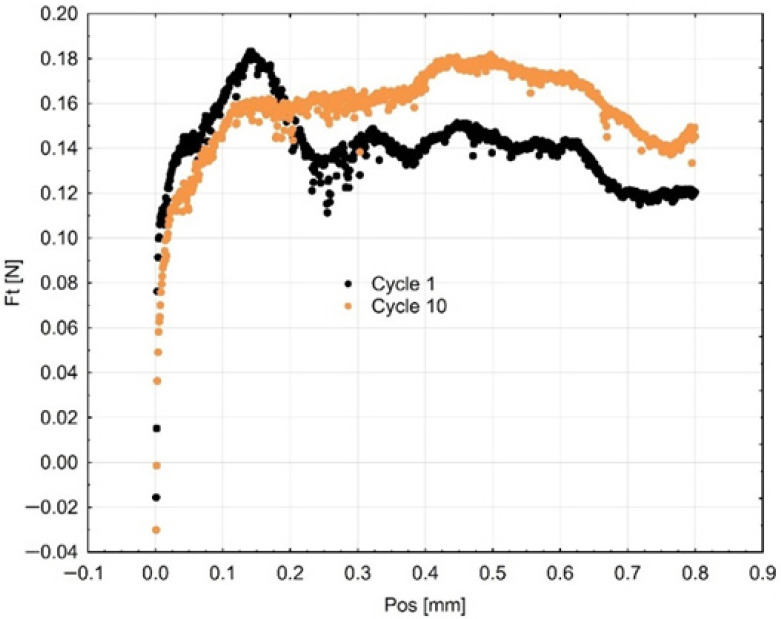
Resistance to scratching (friction force) in the first and tenth cycles on the surface of the Gr-17.1 temporary lt material sample.

The friction force (Fₜ) is used to express the cyclic abrasion resistance in Figure 11, Figure 12 and Figure 13. The friction force fluctuated throughout the successive cycles, reaching its peak at the tenth cycle. The gradual increase in Fₜ may indicate surface hardening or work-hardening after repetitive pressure. This action signifies that the surface layer experiences plastic deformation and a reorganization of the polymer matrix and infill particles, thereby enhancing their resistance to additional penetration by the indenter. The observed trend demonstrates the adaptive tribological response of the investigated materials and their ability to sustain functional integrity under repeated occlusal stresses.

**Figure 14:**
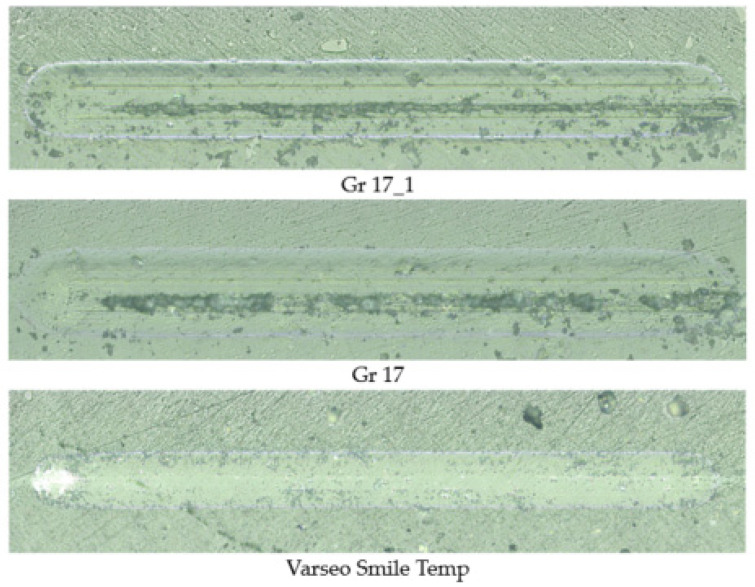
Scratches on the surfaces of biomaterial samples after 10 scratch cycles performed with a Rockwell indenter.

Figure 14 shows selected microscopic pictures of scratches acquired after ten loading cycles. Variations in the mechanisms and severity of the injury are evident in the images. The Gr-17.1 and Gr-17 materials exhibit distinct particle pull-outs in the region affected by the indenter tip, as well as channels at the bottom of the scratch that are oriented parallel to the sliding direction of the indenter. However, the scratches on the surfaces of these materials are significantly broader than those on the VST material. In the case of VST, the scratch groove does not exhibit any cohesive damage after 10 cycles; however, a distinct imprint can be observed at the location of indenter contact.

**Figure 15:**
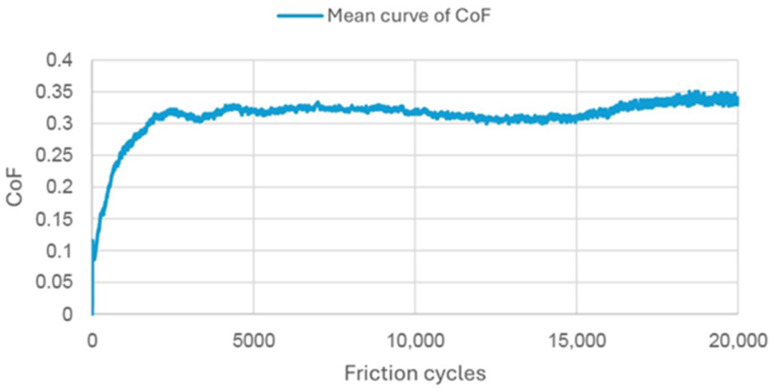
Mean friction coefficient curve for Gr-17.1 material samples.

**Figure 16:**
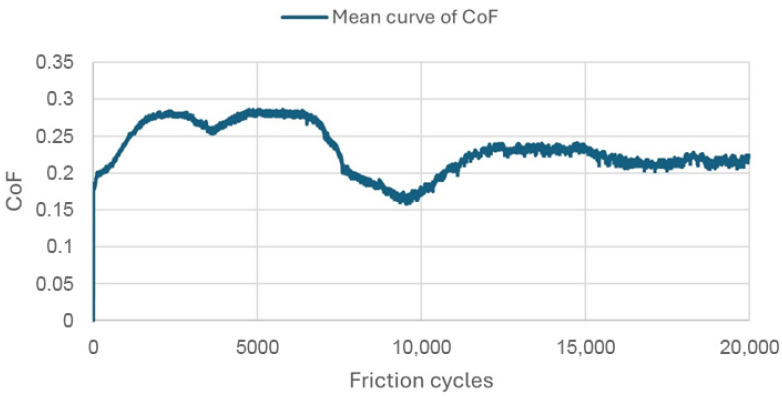
Average friction coefficient curve for Gr-17 material samples.

**Figure 17:**
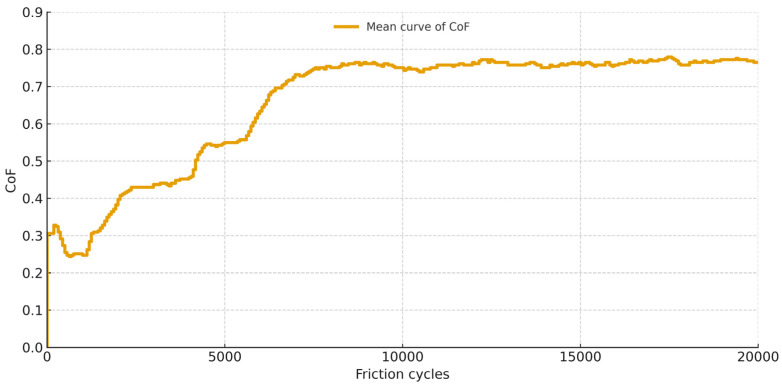
Averaged friction coefficient curve for VST material samples.

The coefficient of friction (CoF) is depicted in Figure 15, Figure 16 and Figure 17 as a function of the number of friction cycles, as determined by experiments conducted in a humid environment at 37 °C. The curves are the average of three tests for each substance. The Gr-17 material demonstrated a multi-stage friction response and the lowest coefficient of friction values, suggesting that the surface progressively adjusted to repeated sliding and that the polymer–filler matrix maintained stability under cyclic loading conditions. In contrast, the highest CoF values were recorded for the VST material, approaching 0.8, indicating greater interfacial adhesion and resistance to sliding.

The data demonstrate that the tribological behavior of each material is significantly influenced by its elastic properties and filler content. This may demonstrate clinical significance regarding occlusal efficiency and wear resistance.

**Figure 18:**
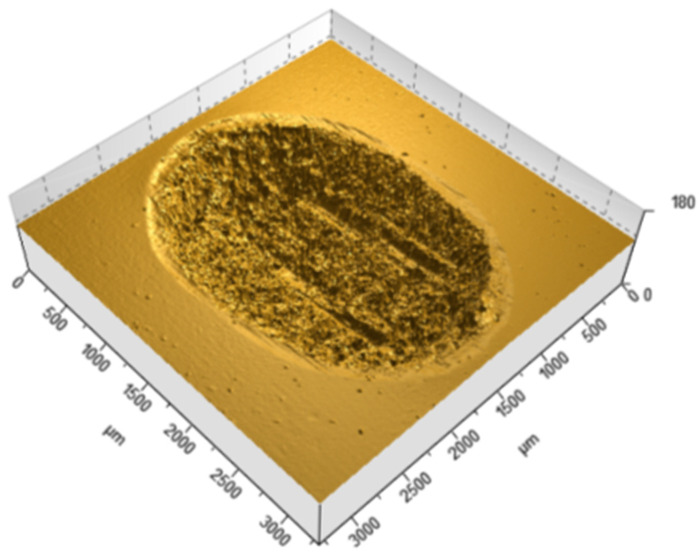
Isometric view of the wear trace on the surface of the Gr-17.1 temporary It material sample.

**Figure 19:**
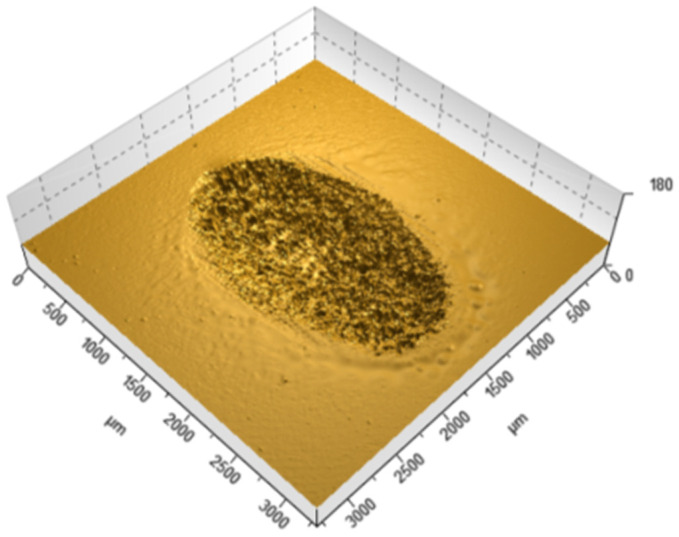
Isometric view of the wear track on the surface of the GR-17 material sample.

**Figure 20:**
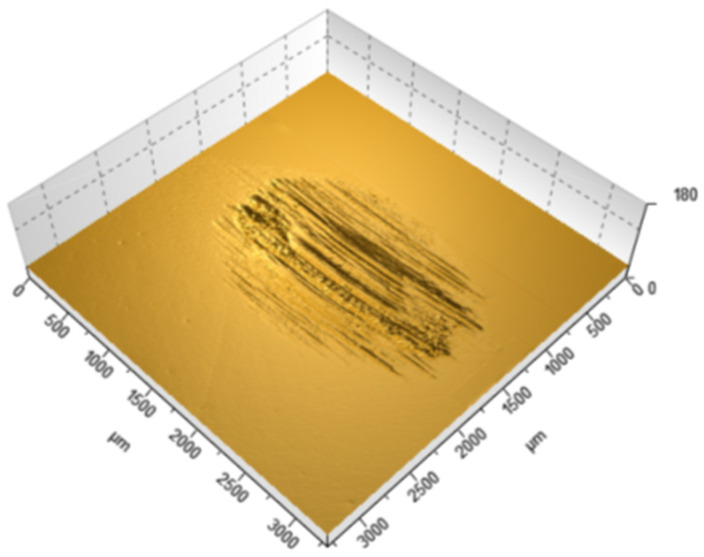
Isometric view of the wear track on the surface of a VarseoSmile Temp material sample.

**Figure 21:**
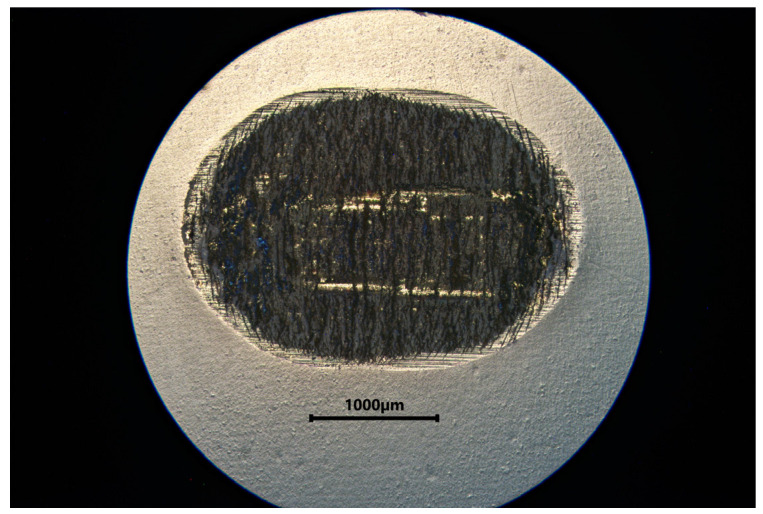
Microphotograph of a tribological wear trace on the surface of a Gr-17.1 material sample.

**Figure 22:**
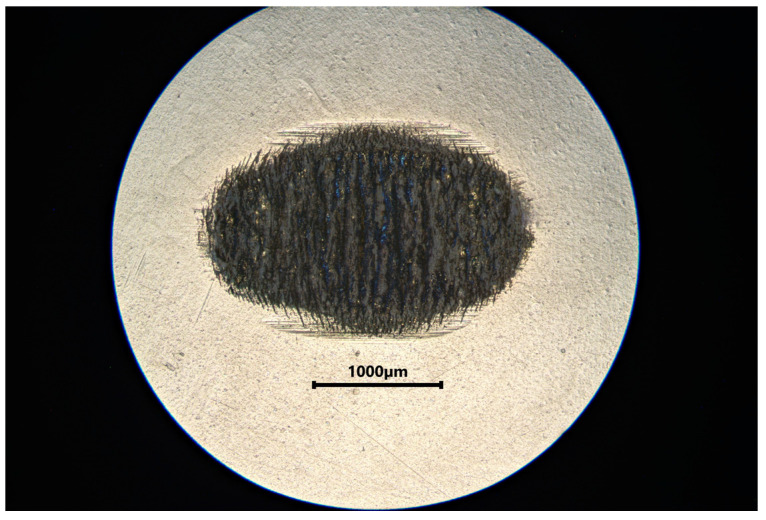
Microphotograph of a tribological wear trace on the surface of a Gr-17 material sample.

**Figure 23:**
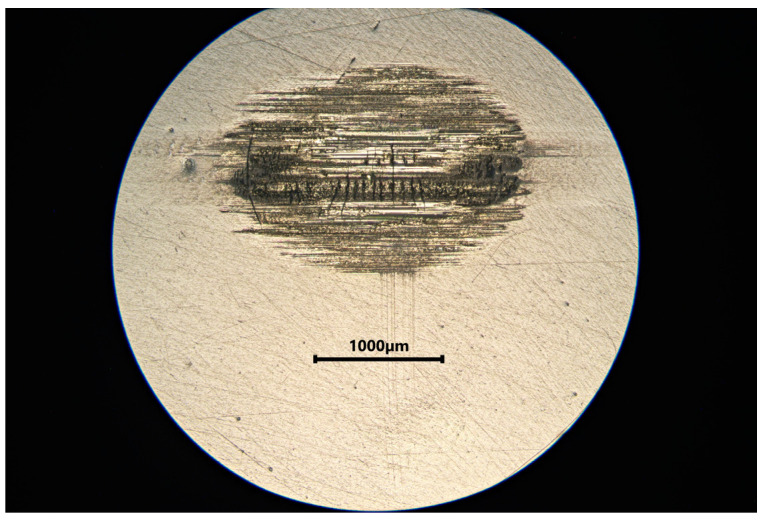
Microphotograph of a tribological wear trace on the surface of a VST material sample.

Table 2 summarizes the wear results, which are illustrated in isometric views in Figure 18, Figure 19 and Figure 20 and images from an optical microscope in Figure 21, Figure 22 and Figure 23. It is intriguing that the coefficient of friction (CoF) did not exhibit a direct positive correlation with volumetric wear. The VST material exhibited the highest stabilized CoF (~0.8) and the lowest volumetric wear. In contrast, Gr-17.1 exhibited the highest wear rate, despite having the most stable and comparatively low CoF (~0.3).

This research suggests that wear resistance is not exclusively determined by frictional coefficient, but is also significantly influenced by microstructural properties, filler content, and material composition. VST’s high CoF and low wear indicate that it has effective load distribution and enhanced surface reinforcement, whereas Gr-17.1’s low CoF and higher wear suggest that it has surface compliance that facilitates material loss and deformation during cyclic loading.

## 4. Discussion

Temporary crowns produced using 3D printing offer significant advantages compared to milling technology, particularly in terms of reducing production time and costs [45]. The dynamic development of additive manufacturing (AM) now enables the production of high-precision, custom-geometry temporary prosthetic restorations for both the anterior and posterior regions [17]. However, during use in the oral cavity, polymeric materials undergo mechanical degradation due to chewing forces, leading to progressive wear and increased surface roughness [30]. Recent findings by Prause et al. [46] indicate that the wear behaviour of 3D-printed dental polymers is strongly dependent on filler–matrix interactions, crosslinking efficiency, and surface homogeneity, which directly influence resistance to microcracking and plastic deformation. These observations are consistent with the present study, where the higher filler content and more uniform internal structure of VarseoSmile Temp resulted in substantially lower volumetric wear and improved surface stability.

The mechanical properties of 3D-printed temporary crowns, evaluated under laboratory conditions, are crucial for determining their clinical suitability [45]. The degree and rate of material damage during treatment are crucial for therapeutic success [20]. Modern C&B materials with fillers allow for the use of temporary crowns for up to 24 months [28]. For this reason, cyclic loading tests were conducted in this study to simulate real operating conditions. The significance of the results obtained in such tests relates to the contact-friction resistance of the surface layer, which plays a fundamental role in transferring biomechanical loads, both physiological and pathological, acting in directions normal and tangential to the occlusal surface [47]. During the chewing action, the vertical movements of the mandible cause bites to be taken, while the horizontal movements allow the lateral teeth to grind and crush food, simultaneously mixing it with saliva. The presence of saliva in the oral cavity is essential for the proper course of this process [47]. For this reason, tribological tests that consider controlled vertical and lateral forces in a humid environment are crucial for a reliable assessment of material properties under preclinical conditions, which is reflected in the test method used here. Additionally, it is proposed to consider the influence of moisture and physiological temperature in tribological studies of C&B materials [48]. Wear resistance under cyclic loading conditions is a characteristic of a complex tribosystem and depends on many factors, including the type of counterface, environmental conditions, and a temperature close to physiological [28,49,50]. The presence of a thin layer of moisture can improve lubrication conditions, but at the same time induce the Rehbinder effect, which influences the wear process [51]. Considering these factors was deemed appropriate in the research method presented here.

Tribological analysis of the research conducted showed that the tested materials differ in their wear resistance. This differentiation can result from differences in the structural composition and filler content within the polymer matrix. The filler additive increases the surface hardness of the material and limits mass loss during sliding contact [52]. Materials Gr-17.1 and Gr-17 contained silica particles up to 2% *w*/*w*, while VarseoSmile Temp (VST) material contained silanized dental glass, with a total inorganic filler content of 30–50% *w*/*w* and an average particle size of 0.7 μm. The VST material, characterized by the highest filler content, exhibited the highest wear resistance, as confirmed by both the lowest volumetric wear values (Table 2) and the lowest permanent scratch depth. The works [53,54,55] demonstrated that the wear of polymer dental composites depends on the filler content and size in the matrix. Generally, it can be assumed that the presence of nanoparticles in the structure of the surface layer strengthens and homogenizes it and slows down the wear process [56,57]. Furthermore, smaller filler particles may favorably influence load transfer from the matrix to the filler [58]. Smaller filler sizes result in a larger surface area-to-volume ratio, which leads to better interaction, including force interaction, between the filler and the matrix [11]. High wear resistance is clinically significant as it allows for the maintenance of vertical dimensions during the use of temporary crowns [28].

The material structure also influences the dominant wear mechanisms. In the case of Gr-17.1 material, which showed the highest wear, the dominant mechanism was likely fretting, characteristic of sliding-oscillating motion [59]. Fretting wear in polymeric materials is understood as surface degradation caused by small-amplitude, reciprocating micro-movements that induce repeated local slip, micro-fatigue, and progressive surface fragmentation. This mechanism is typically identified by irregular shallow depressions, micro-pits, and debris accumulation resulting from cyclic disruption of weakly supported surface layers [60]. The worn areas of the Gr-17.1 samples exhibited several of these features, which is consistent with fretting-driven degradation. In contrast, regular, narrow grooves aligned with the direction of the friction vector were observed in the VST material (Figure 18). A similar phenomenon was described in work [61]—the formation of narrow grooves with local cracks and delamination, which indicates stronger intralayer bonds and the effect of filler reinforcement.

The VST material was characterized by a high friction coefficient while maintaining low wear. Friction coefficient values are clinically significant because too high a value can lead to premature wear, while too low a value can reduce the effectiveness of food breakdown [60]. The wear coefficient allows for the assessment of the kinetic and energetic aspects of material interaction during chewing and their tribological resistance [62]. The stepwise increase in the friction coefficient of C&B materials after a certain number of cycles can be the result of the formation of wear products that change the contact nature to three-body and promote the formation of a tribofilm [62,63].

The most stable friction coefficient was exhibited by the Gr-17.1 lt material (Figure 13), in which stabilization occurred after approximately 2000 friction cycles. For the VST material, the stabilization of the friction interaction was noted after approximately 7000 cycles, while for Gr-17, it was only after several thousand cycles.

The results of the incremental scratch resistance (Rd) tests showed significant differences between the VST material, and the other composites tested. Materials with a higher filler content were characterized by smaller scratch sizes. Differences compared to the results obtained under sliding friction conditions may be due to the interaction of the indenter with small filler particles [29]. When using a Rockwell indenter, the Hertz contact area is only about five times larger than the average size of even such small particles as nanofillers, which increases the probability of contact with individual particles [64]. However, in materials Gr-17.1 and Gr-17, the low filler content limits this effect.

In a tribological context, new 3D-printed materials should exhibit wear resistance comparable to conventional materials to ensure the stability of occlusal contacts and the long-term functionality of temporary crowns [65,66,67]. Lower resistance can lead to a shorter lifespan of restoration and premature development of cavities on occlusal surfaces. Appropriate shaping of the material structure characteristics, particularly the optimization of the inorganic filler content, can significantly improve the durability and tribological resistance of temporary prosthetic restorations.

## 5. Conclusions

Based on the obtained results and analyses, the following conclusions were drawn:

1. Wear resistance strongly depends on the composition and microstructure of the material. The resin with the highest inorganic filler content (VST, 30–50 wt%) demonstrated the lowest volumetric wear, the shallowest scratch depth, and the most stable surface structure. In contrast, Gr-17 and Gr-17.1, which contain significantly lower filler content, exhibited higher surface degradation and reduced mechanical stability.
2. The coefficient of friction does not correlate directly with wear intensity. Despite the highest stabilized friction coefficient (~0.8), VST showed the lowest wear, whereas Gr-17.1, with a considerably lower friction coefficient (~0.3), experienced greater material loss. This indicates that microstructural reinforcement and filler–matrix interactions play a more decisive role in wear resistance than friction alone.
3. Scratch resistance is closely linked to filler distribution and elastic recovery. VST showed the greatest resistance to plastic deformation under both incremental and cyclic scratch loading, confirming its superior surface elasticity and reinforcing the importance of filler-based strengthening in temporary crown materials.
4. Tribological evaluation under physiological conditions is essential for predicting clinical performance. Testing in a humid environment at 37 °C and under cyclic loading provides relevant insight into the behavior of temporary crowns during mastication and supports the suitability of such methods for preclinical assessment.
5. Material optimization based on preclinical testing can significantly improve the durability and dimensional stability of temporary restorations. Selecting materials with enhanced filler content and more homogeneous microstructures may help maintain occlusal integrity and functional properties throughout the intended treatment period.
6. Future studies should incorporate advanced microstructural characterization techniques (e.g., EDS, µCT, high-resolution interface analysis) to further investigate the role of filler–matrix interactions in wear behavior. The development of novel photopolymer resins with increased and diversified filler phases, as well as optimization of DLP printing parameters such as layer thickness, build orientation, and exposure settings, could contribute to further improvements in tribological and mechanical performance.

## Figures and Tables

**Table 1:** Characteristics of the materials used in the comparative study.

Parameter	Gr-17.1 Temporary It (Abbrev. Gr-17.1)	Gr-17 Temporary (Abbrev. Gr-17)	VarseoSmile Temp (Abbrev. VST)
Manufacturer	Pro3dure	Pro3dure	BEGO
3D printer and parameters	ASIGA UV MAX (wavelength 385 nm, 1919 dpi × 1081 dpi, 62 µm)	ASIGA UV MAX (wavelength 385 nm, 1919 dpi × 1081 dpi, 62 µm)	ASIGA UV MAX (wavelength 385 nm, 1919 dpi × 1081 dpi, 62 µm)
Manufacturer’s description (intended use)	Biocompatible material for temporary crowns and bridges; long-term dental restorations (anterior and posterior region), prosthetic teeth.	Biocompatible material for temporary crowns and bridges used in the anterior region.	Biocompatible material for temporary crowns and bridges; prosthetic restorations for anterior and posterior regions.
Resin	7,7,9(or 7,9,9)-trimethyl-4,13-dioxo-3,14-dioxa-5,12-diazahexadecane-1,16-diyl bismethacrylate,	7,7,9(or 7,9,9)-trimethyl-4,13-dioxo-3,14-dioxa-5,12-diazahexadecane-1,16-diyl bismethacrylate,	“4,4′-isopropylidiphenol, ethoxylated, and 2-methylprop-2-enoic acid; silanized dental glass; methyl benzoylformate; diphenyl (2,4,6-trimethylbenzoyl) phosphine oxide.”
	3,6,9-trioxaundecamethylene Dimethacrylate,	3,6,9-trioxaundecamethylene Dimethacrylate,	
	Phenyl bis(2,4,6-trimethylbenzoyl)-phosphine oxide	Phenyl bis(2,4,6-trimethylbenzoyl)-phosphine oxide	
Filler	Contain inorganic fillers with a particle size in the range of 0.4–3 μm, filler content of ~40.0 m-%.	Contain inorganic fillers with a particle size in the range of 0.4–3 μm, filler content of ~20.0 m-%.	Silanized glass-ceramic filler with a declared content of approximately 30–50 wt% (average particle size ~ 0.7 μm).
Elastic modulus	5528 MPa [37]	2442 MPa [37]	≥3500 MPa
Flexural strength	169 MPa [38]	113 MPa [37]	≥100 MPa
Shore D hardness	80 ShD [39,40]	80 ShD [39]	≥90 ShD [41]

**Table 2:** Wear of tested materials.

Materials	Gr-17.1 Temporary It	Gr-17 Temporary	VarseoSmile Temp
	Wear [mm^3^]		
Sample 1	0.190174	0.064141	0.026421
Sample 2	0.262957	0.071113	0.018415
Sample 3	0.251218	0.073901	0.030516
Mean	0.234783	0.069718	0.025117

## Data Availability

The original contributions presented in this study are included in the article. Further inquiries can be directed to the corresponding author.

## Abbreviations

The following abbreviations are used in this manuscript:

AM	Additive Manufacturing
BEGO	BEGO GmbH (manufacturer of VarseoSmile Temp)
C&B	Crown and Bridge
CoF	Coefficient of Friction
DLP	Digital Light Processing
ISO	International Organization for Standardization
MST	Micro Scratch Tester
Pd	Penetration Depth
Rd	Residual Scratch Depth
RH	Relative Humidity
ShD	Shore D Hardness
SRV	Schwingung Reibung Verschleiß (Oscillating Friction and Wear tester)
UV	Ultraviolet
VST	VarseoSmile Temp
WLI	White Light Interferometry
